# Supplementary material for: Identification of photoperiod-induced specific miRNAs in the adrenal glands of Sunite sheep (Ovis aries)
Source: Front Vet Sci. 2022 Jul 22;9:888207. doi: 10.3389/fvets.2022.888207 (PMC9354845; doi:10.3389/fvets.2022.888207)
Supplement: Supplementary file 1 [file Data_Sheet_1.ZIP › Supplementary file/miRDeep2 file.zip!/miRDeep2 file/Know_Structure/LP42A3/oar-mir-27a.pdf]

miRBase precursor : oar-mir-27a
Total read count : 259403
oar-miR-27a read count : 259208
remaining reads : 195

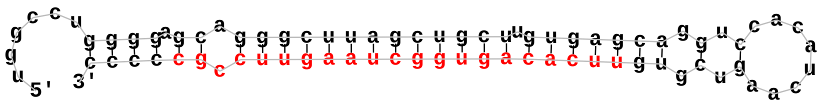

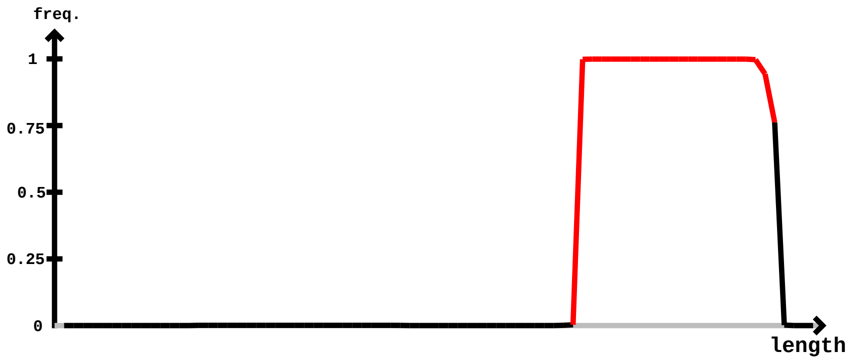

oar-miR-27a

```
5'- uggccuggggagcagggcuuagcugcuugugagcagguccacaucaagucguguucacaguggcuaaguuccgccccc  -3'  exp
    ......((((.((.(((((((((((((.(((((((.(.(........).).))))))))))))))))))))).))))))   reads   mm   sample
    ............agggcuuagcugcuugugagc.............................................   31      0    seq
    ............agggcuuagcugcuugugagca............................................   152     0    seq
    ............agggcuuagcugcuugugagcag...........................................   1       0    seq
    ............agggcuuagcugcuugugagcagg..........................................   7       0    seq
    ............agggcuuagcugcuugugagcaggu.........................................   4       0    seq
    ...................................................uguucacaguggcuaagu.........   1       0    seq
    ...................................................uguucacaguggcuaaguuc.......   6       0    seq
    ...................................................uguucacaguggcuaaguucc......   20      0    seq
    ...................................................uguucacaguggcuaaguucU......   4       1    seq
    ...................................................uguucacaguggcuaaguuccg.....   173     0    seq
    ...................................................uguucacaguggcuaaguucUg.....   1       1    seq
    ...................................................uguucacaguggcuaaguuccgc....   27      0    seq
    ...................................................uguucacaguggcuaaguuccgcc...   1       0    seq
    ....................................................guucacaguggcuaaguucU......   28      1    seq
    ....................................................guucacaguggcuaaguucc......   5       0    seq
    ....................................................guucacaguggcuaaguucUg.....   81      1    seq
    ....................................................guucacaguggcuaaguucUgc....   421     1    seq
    .....................................................uucacagugUcuaaguu........   1       1    seq
    .....................................................uucacaguggcuaaguu........   39      0    seq
    .....................................................uucacaguggcuaaguG........   1       1    seq
    .....................................................uucacaguggcuaagCu........   1       1    seq
    .....................................................uucacaguggcuaaguuG.......   1       1    seq
    .....................................................uucacagugUcuaaguuc.......   1       1    seq
    .....................................................uucacaguggcuaaguuc.......   508     0    seq
    .....................................................uucacaguAgcuaaguuc.......   1       1    seq
    .....................................................uucacaguggcuaaguucU......   11412   1    seq
    .....................................................uucacaguggcuaaguucc......   2418    0    seq
    .....................................................uucacaguggcuaaguuccA.....   6219    1    seq
    .....................................................uucacaguggcuaaguucUg.....   20034   1    seq
    .....................................................uucacaguggcuaaguuccg.....   20589   0    seq
    .....................................................uucacaguggcuaaguucUgc....   191216  1    seq
    .....................................................uucacaguggcuaaguuccgc....   5324    0    seq
    .....................................................uucacaguggcuaaguuccgcc...   453     0    seq
    .....................................................uucacaguggcuaaguuccgccc..   6       0    seq
    ......................................................ucacaguggcuaaguucc......   3       0    seq
```

oar-miR-27a

```
uggccuggggagcagggcuuagcugcuugugagcagguccacaucaagucguguucacaguggcuaaguuccgccccc
......................................................ucacaguggcuaaguucU......    8       1       seq
......................................................ucacaguggcuaaguuccA.....    5       1       seq
......................................................ucacaguggcuaaguucUg.....    17      1       seq
......................................................ucacaguggcuaaguuccg.....    20      0       seq
......................................................ucacaguggcuaaguuccgc....    6       0       seq
......................................................ucacaguggcuaaguucUgc....    133     1       seq
.......................................................cacaguggcuaaguucU......    1       1       seq
.......................................................cacaguggcuaaguucUg.....    2       1       seq
.......................................................cacaguggcuaaguuccg.....    3       0       seq
.......................................................cacaguggcuaaguucUgc....    16      1       seq
........................................................acaguggcuaaguucUg.....    1       1       seq
........................................................acaguggcuaaguucUgc....    1       1       seq
```
